# The Role of Cytokines in the Different Stages of Hepatocellular Carcinoma

**DOI:** 10.3390/cancers13194876

**Published:** 2021-09-29

**Authors:** Noe Rico Montanari, Chimaobi M. Anugwom, Andre Boonstra, Jose D. Debes

**Affiliations:** 1 Department of Medicine, Division of Gastroenterology & Division of Infectious Disease, University of Minnesota, Minneapolis, MN 55455, USA; n.ricomontanari@erasmusmc.nl (N.R.M.); anugw001@umn.edu (C.M.A.); 2 Department of Gastroenterology and Hepatology, Erasmus MC, 3015 CE Rotterdam, The Netherlands; p.a.boonstra@erasmusmc.nl; 3 Health Partners Digestive Care, Saint Paul, MN 55130, USA

**Keywords:** cytokines, hepatocellular carcinoma, prognosis, formation, advanced disease, response to therapy

## Abstract

**Simple Summary:**

Non-homeostatic cytokine expression during hepatocellular carcinogenesis, together with simple and inexpensive cytokine detection techniques, has opened up its use as potential biomarkers, from cancer detection to prognosis. However, carcinogenic programs during cancer progression are not linear. Therefore, cytokines with prognostic potential in one stage may not be relevant in another. Here, we reviewed cytokines with clinical potential in different settings during hepatocellular carcinoma progression.

**Abstract:**

Hepatocellular carcinoma (HCC) is the primary form of liver cancer and a leading cause of cancer-related death worldwide. Early detection remains the most effective strategy in HCC management. However, the spectrum of underlying liver diseases preceding HCC, its genetic complexity, and the lack of symptomatology in early stages challenge early detection. Regardless of underlying etiology, unresolved chronic inflammation is a common denominator in HCC. Hence, many inflammatory molecules, including cytokines, have been investigated as potential biomarkers to predict different stages of HCC. Soluble cytokines carry cell-signaling functions and are easy to detect in the bloodstream. However, its biomarkers’ role remains limited due to the dysregulation of immune parameters related to the primary liver process and their ability to differentiate carcinogenesis from the underlying disease. In this review, we discuss and provide insight on cytokines with clinical relevance for HCC differentiating those implicated in tumor formation, early detection, advanced disease, and response to therapy.

## 1. Introduction

Liver cancer is a leading cause of cancer-related death worldwide with approximately 800,000 deaths per year, with hepatocellular carcinoma (HCC) representing the great majority of primary liver cancers [1,2,3]. Epidemiological data have shown marked differences in HCC incidence among different ethnic-racial groups, genders, and across geographic regions of the globe, partially dictated by different risk factors. Among the main risk factors are infection with the hepatitis B virus (HBV) or hepatitis C virus (HCV) and alcohol use [4]. Irrespective of the different etiologies, unresolved chronic inflammation is a common denominator and a feature present in more than 90% of patients with HCC [5]. Local activation of cell populations upon sensing pathogens and/or tissue damage in the liver may trigger a tightly regulated and coordinated multi-step process, followed by immune cell infiltration, and subsequent engagement in tissue repair as the ultimate goal [6]. It is in this fine orchestration of events that the release of a wide array of soluble factors, such as cytokines, takes place [7].

**Figure 1:**
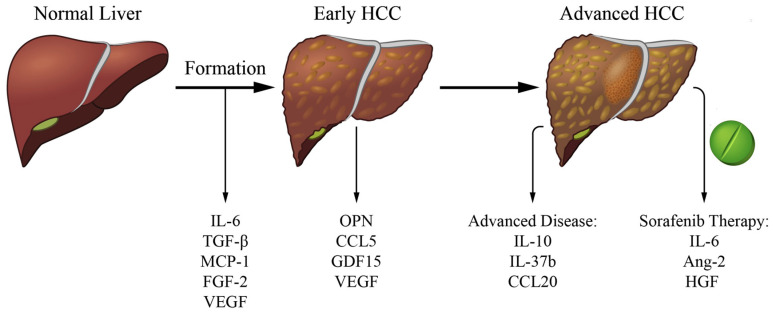
Cytokines of clinical relevance in the different stages of liver cancer. List of selected cytokines involved in tumor formation, relevant in early HCC detection and with prognosis potential in advanced disease and response to systemic (sorafenib) therapy.

In this regard, cytokines have been investigated as potential biomarkers to predict different stages of HCC, and to further understand mechanisms of HCC formation. In the presence of HCC-promoting risk factors, the initial inflammatory response in the liver is unresolved, and as a result, the unbalanced expression of cytokines promotes a persistent healing response. This response may lead to sequential development of fibrosis, cirrhosis, and eventually HCC by enhancing hepatocyte proliferation and regeneration which can lead to mutagenesis and set the stage for HCC development [8]. Once HCC is established, cytokines released by the tumor, neighboring non-tumor cells, or immune cells can act on the malignant lesion to promote tumor survival by multiple mechanisms [9,10]. In addition, these cytokines can act on the tumor microenvironment to induce immune escape and metastasis [11]. Interestingly, as the treatment of advanced HCC has evolved from no reasonable therapy to tyrosine kinase inhibitors that significantly prolong survival to immune therapy, cytokines can act as markers of response to therapy [12,13]. Since cytokines are present throughout the different stages of HCC progression, their evaluation may provide insightful information on HCC detection and management. The ability to detect cytokines in sera and/or plasma could potentially serve as biomarkers to increase early HCC detection rates which would improve disease outcome as well as be used as prognostic factors in response to therapies [14,15]. It is important to highlight, however, that certain cytokines—although involved in a common carcinogenic program, such as angiogenesis—might more accurately depict a given stage in HCC progression than others, and that cytokines with prognostic potential in one stage may not be relevant in another. In this review, we focus on selected cytokines that are not only relevant to tumor formation, but also to clinical progression and potential prognostic value in early HCC detection as well as in response to therapy (Figure 1). To note, here we only included what those cytokines we interpreted to be the most significant either based on 3 or more manuscripts showing implication in the role or a highly significant manuscript. In addition, we chose cytokines that are easily measurable in peripheral blood (which would exclude EGF, wnt-b-catenin).

## 2. Cytokines Related to HCC Formation

**Figure 2:**
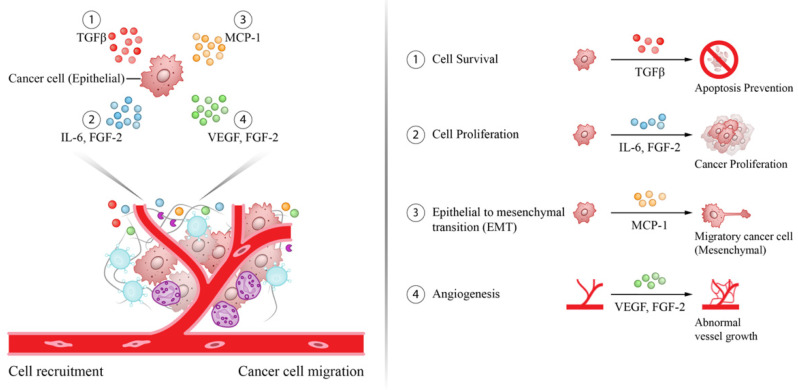
Pro-carcinogenic cytokines in the tumor microenvironment involved in HCC formation. Effect of selected cytokines in the tumor microenvironment contributing to HCC formation by promoting cancer cell survival (TGF-β), proliferation (IL-6, FGF-2), epithelial mesenchymal transformation (MCP-1), and angiogenesis (VEGF, FGF-2).

Due to its physiologic role and anatomic location the liver is exposed to chronic infections and environmental insults resulting in an unresolved inflammation state that may lead to HCC. It is in this setting that the presence of pro-inflammatory cytokines in peritumoral tissues contributes to tumor formation as well as progression. Most of these cytokines participate in carcinogenesis by inducing cell survival and proliferation, epithelial mesenchymal transition (EMT), and angiogenesis (Figure 2).

### 2.1. Interleukin-6 (IL-6)

One of the cytokines most frequently examined in both mouse and human studies with respect to the early stages of cancer formation is IL-6. This pro-inflammatory cytokine has a critical role in host defense and in the orchestration of inflammation leading to cancer [16,17,18]. In HCC, the constant exposure to triggering insults in the liver (i.e., during chronic viral hepatitis infection, or alcohol use) leads to a chronic inflammatory state that eventually promotes cancer formation [19]. In human studies, increased levels of serum IL-6 in HCC patients -compared to chronic hepatitis and cirrhosis patients- have been consistently shown [20]. Furthermore, among HCC patients, IL-6 levels have been found to be increased in advanced vs. early stages of HCC supporting the conception of IL-6 as an important cytokine in hepatocarcinogenesis [21,22]. Moreover, it has been shown that elevated serum IL-6 levels in HCC patients who undergo hepatectomy (*n* = 144) are associated with lower overall survival and experience early HCC recurrence [23].

In vivo experiments performed in a diethylnitrosamine (DEN) HCC mouse model with hepatocyte-specific knockout of the IL-6 receptor gp130 have demonstrated a reduced number of liver tumor nodules and macrophages compared to their control counterparts, supporting a role for IL-6 in HCC formation [24]. Moreover, Kupffer cells, the macrophages of the liver, can act as source of IL-6 upon stimulation with the microbial product lipopolysaccharide, which supports pre-malignant hepatocyte proliferation under DEN-induced carcinogenesis [25]. Of note, increased serum IL-6 levels have been found in cirrhotic patients without HCC compared to healthy controls which may be the consequence of increased microbial translocation, commonly observed in cirrhotic patients [26]. Interestingly, estrogen-mediated inhibition of IL-6 production by activated Kupffer cells reduced chemical hepatocarcinogenesis in DEN-HCC mice and has been proposed as a mechanism behind sex disparities in HCC [27]. Similarly, IL-6 blockade in multidrug resistance 2 knockout mice showed decreased liver carcinogenesis [28]. This effect likely occurred due to a decrease in hepatocytes harboring genomic instability, instated by a genotoxic environment, which reinforces a role of IL-6 in promoting survival of pre-malignant hepatocytes [28]. Furthermore, mice models have shown the immune-suppression role of IL-6 by inducing PD-L1 expression on tumor-associated macrophages, which are associated with immune-evasion [29]. Lastly, studies in mouse HCC models have demonstrated that isolated HCC progenitor cells can give rise to cancer when there is ongoing liver damage, and that these cells promote their own growth and progress towards malignancy via autocrine IL-6 signaling [30].

### 2.2. Transforming Growth Factor Beta (TGF-β)

The cytokine TGF-β regulates many inflammatory processes, which generally lead to inhibition of cellular processes, such as proliferation, differentiation, and survival [31]. Since the TGF-β receptors (TGF-βR) are broadly expressed, TGF-β can act on virtually all cells. The TGF-βR heterodimer consists of 2 chains which upon triggering, activates SMAD-dependent signal transduction cascades to induce gene expression of the target genes [31]. During carcinogenesis, malignant cells can often blunt their suppressive TGF-β signaling by altering the expression of its receptors, but also hijack the signaling cascade to inactivate growth-inhibitory functions [31]. In HCC, mutations have been described in the TGFBRII poly(A) region of the gene, which were found to encode for non-active receptors [32]. Moreover, HCC cell lines with metastatic potential have been described to downregulate TGF-βR2. Interestingly, reduced TGF-βR2 expression in HCC tissues was found to correlate with larger tumor size and various metastatic features, such as poor differentiation, portal vein invasion and intrahepatic metastasis [32]. Moreover, mutations in SMAD2 and SMAD4 genes have been observed in HCC which can result in cell cycle progression via disruption of cyclin inhibitors, such as p15INK4b and p21CIP1 [33,34,35,36]. Furthermore, methylation of the cyclin inhibitors p16INK4a and p15INK4b is an event found in early stages of HCC as well as in cirrhotic patients, although at a smaller rate, suggesting that these epigenetic modifications play a role in certain aspects of hepatocarcinogenesis [37,38,39]. Interestingly, non-canonical SMAD-independent signal transduction via TAK1—also known as mitogen-activated protein kinase 7—can activate p38 and JNK kinases, which are known to participate in HCC [40,41]. Upon JNK activation, a non-canonical SMAD3 isoform (pSmad3L) becomes active, resulting in silencing of signals of cell cycle arrest and augmented cell proliferation [42]. In contrast, JNK inhibition has been shown to reduce HCC tumors in a DEN-HCC rat model [43]. Interestingly, immunostaining of oncogenic JNK signaling molecules in livers of chronic HBV patients was found to be increased during progression from cirrhosis to HCC [44]. Similar results were found in HCV-induced HCC livers as fibrotic and necro-inflammatory grades progressed [45]. Moreover, TGF-β signaling has been shown to induced surface tumor associated markers (i.e., CD133 and CD90) in liver progenitor cells which coffered them tumor intrinsic cell properties such as, increased self-renewal potential and greater chemoresistance potential [46]. A proposed mechanism for the increased chemoresistance potential was recently proposed where TGF-β induced the expression drug-efflux transporters via the induction of the xenobiotic nuclear receptor, PXR [47].

### 2.3. Monocyte Chemoattractant Protein 1 (MCP-1)

Produced by parenchymal and non-parenchymal liver cells upon tissue injury, MCP-1 acts as a potent chemoattractant of immune cells by interacting with the CC chemokine receptor 2 (CCR2) [48]. In HCC mouse models increase in MCP-1 expression plays a pivotal role in the recruitment of monocyte-derived macrophages [49,50]. In the tumor microenvironment, these cells can support dysplastic lesions by promoting angiogenesis and cancer cell proliferation by the release of metalloproteinases (MMPs) and cytokines such as IL-6 and TGF-ß. In addition, these macrophages also suppress effective anti-tumor immune responses by limiting antigen presentation and inducing immunotolerance in favor of the tumor [51,52]. Further illustrating the relevance of MCP-1 in relation to macrophages, it was shown that CCR2 antagonists inhibit HCC growth in an orthotopic mice model where murine hepatoma cells were implanted in the liver [53]. This outcome was accompanied by a reduction of recruited pro-tumorigenic monocytes and an increase of anti-tumor cytotoxic CD8 T cells. In line with this, human HCC livers with increased MCP-1 expression show a higher numbers of macrophages and reduced CD8 T cell numbers in the tumor [53]. On the other hand, laboratory assays have shown MCP-1 to promote migration and invasion in hepatoma-lines (i.e., Huh7 and Hep3B) by downstream activation of activating protein-1 (AP-1) which in turn induces the onco-microRNA miR-21 promoting cancer cell migration and invasion [54].

MCP-1-stimulated HCC cell lines also showed an EMT phenotype which encompassed morphological changes with increased expression of stem markers (i.e., N-cadherin, vimentin) and enhanced metastatic potential when transplanted into nude mice [54]. Interestingly, human data on MCP-1 have shown an increase in the number of MCP-1—expressing endothelial progenitor cells—associated with advanced HCC stages and have been hypothesized to promote neo-vascularization by promoting angiogenesis via release of pro-angiogenic cytokines [54].

### 2.4. Vascular Endothelial Growth Factor (VEGF)

The role of VEGF as an angiogenic and tumorigenesis factor has been known for almost three decades and has been extensively reviewed elsewhere [55,56]. Under normal liver homeostasis, VEGF is predominantly expressed by hepatic stellate cells and myofibroblast at low levels [56,57]. In contrast, during HCC formation and progression, VEGF expression by these cells in human livers is increased [58]. Oxidative stress, hypoxia, and nutrient deprivation are hallmarks of tumor formation and have been shown to stimulate VEGF expression [59,60,61,62]. Interestingly, malignant hepatocytes in human HCC tumors have been shown to expressed higher cytoplasmatic VEGF levels than non-malignant hepatocytes located in cirrhotic areas [62].

As an angiogenic factor, VEGF induces new vessel formation, which can act as new ports for the recruitment of inflammatory cells, inducing further inflammation. In addition, new vessels may act as exit windows for tumor cells to gain access to the circulation to metastasize46. Interestingly, the lack of well-defined vessel architecture can offer sub-optimal oxygen and nutrient supply, which may select for more aggressive forms of tumors, while increasing hepatocyte damage and hypoxia46. All of these factors play a critical role in hepatocarcinogenesis. As a liver nodule transitions to a tumor, the so-called “portal triad” becomes less frequent and “unpaired arteries” become the norm. It is in this setting that VEGF promotes HCC neovascularization [63].

### 2.5. Fibroblast Growth Factor 2 (FGF-2)

FGF-2 has been shown to be expressed in human tumors since the late 80s and early in vitro work on hepatoma cell lines demonstrated that almost all cells express FGF-2 at the mRNA level [64]. Importantly, exogenous FGF-2 can induce cell proliferation rendering this cytokine an attractive target in HCC therapy [65]. FGF-2 neutralization with monoclonal antibodies in HCC xenograft mouse models has demonstrated reduced tumor growth [66]. FGF-2’s mode of action is not limited to cell proliferation, but has also been indirectly linked to tumor angiogenesis. This was demonstrated using a double-chamber in vitro assay in which FGF-2 secreted by hepatoma cells induced T-cadherin, an adiponectin related to neovascularization, on liver sinusoidal endothelial cells [67]. Interestingly, T-cadherin expression is often observed in intra-tumoral capillary endothelial cells in HCC tissues, but not in liver control tissues [68]. Moreover, serum FGF-2 levels are increased during progression of chronic liver disease and correlate with large tumors (>5 cm), with the presence of venous invasion and with advanced TNM stage, suggesting a role for FGF-2 in HCC angiogenesis progression [69,70]. 

## 3. Cytokines Linked to Early Detection

Early detection of HCC remains the best tool in HCC management as curative treatment at this stage achieves the highest survival rates of patients. However, ultrasound surveillance for HCC detection—the standard approach for patients at risk—estimates a pooled 45% sensitivity for early HCC detection by a recent meta-analysis [71]. An attractive option to replace ultrasound, is the use of blood biomarkers as they are easily quantifiable and interpretable through standardized assays. In this section, we aim at describing serum or plasma cytokines with potential clinical use.

### 3.1. Osteopontin (OPN)

OPN has been examined as an early HCC marker by many research groups. OPN is highly expressed at sites of inflammation and tissue remodeling and can be produced by Kupffer cells, hepatic stellate cells, and hepatocytes [72,73,74]. This cytokine mediates a wide array of biological functions in the immune and vascular system and has been studied extensively in numerous cancers [75]. Increased serum and plasma levels of OPN in individuals with HCC compared to those with liver cirrhosis or chronic liver disease controls have been reported in several studies [76,77,78,79,80,81,82]. Most of these studies were dominated by Asian cohorts albeit these findings were also true in a West African and European cohort [79,82]. Moreover, the diagnostic performance of OPN discriminating HCC from non-HCC, reported as area under the curve (AUC), was 0.75 or higher in most studies with one exception which may be explained by the inclusion of non-viral etiologies (i.e., NASH and alcohol) [79]. Despite promising results for HCC vs. non-HCC, the specific diagnostic efficacy of OPN in detecting early stage HCC from non-HCC patients varies considerably depending on the study. Evaluation of OPN levels in patients with early stage HCC (Barcelona Clinic Liver Classification, BCLC, stage 0-A) resulted in an AUC value for OPN of 0.57 and 0.78, and another study reported an AUC of 0.73 in BCLC stage A HCC patients [76,78,79]. Furthermore, Zhu et al. reported an impressive AUC of 0.86 discriminating small HCC (<2 cm) vs. non-HCC [80]. Interestingly, a prospective evaluation in an Asian cohort of 115 chronic liver disease patients (mainly viral) at risk of HCC showed increased plasma OPN levels 24 months prior to HCC diagnosis in 21 subjects [82]. These findings were later reproduced in the European Prospective Investigation into Cancer and Nutrition (EPIC) cohorts. In a similar fashion as the Asian study, EPIC found that OPN levels within 2 years of diagnosis had a reasonable HCC predictive value with an AUC of 0.82 [83].

### 3.2. CC Chemokine Ligand 5 (CCL5)

CCL5 is a chemoattractant of memory T cells and other immune cell types, which has been shown to be critical in controlling chronic viral infections [84]. CCL5 has also been shown to be associated with liver inflammation in the setting of chronic HCV and HBV as well [85,86]. To date, only one study, in a European setting, has evaluated serum CCL5 levels in the context of HCC detection. This study examined 61 HCC cases compared to 78 controls and found increased serum CCL5 levels in HCC patients [87]. A multivariate forward stepwise regression analysis associated CCL5 levels higher than 0.86 ng/mL to occurrence of HCC (Odds ratio = 3.63) [87]. Moreover, CCL5 performance in HCC detection had an AUC of 0.72 with a sensitivity (71%) and specificity (68%) [87]. To our knowledge, no other study has yet reproduced these findings in a different cohort of patients.

### 3.3. Growth Differentiation Factor 15 (GDF15)

A divergent member of the TGF-β superfamily, GDF15, is rarely detected under homeostatic conditions, except in human placenta where it is abundant [88]. Increased levels of this marker are observed in pathological conditions such as inflammation, ischemia, and some forms of cancer [88]. In the context of HCC, comparison of serum GDF15 levels in a Chinese cohort of 223 HCC cases, predominantly due to viral hepatitis, showed elevated levels in sera of HCC patients as compared to HBV/HCV controls [89]. Importantly, although serum GDF15 levels were increased in HCC patients compared to chronic HBV and HCV, no statistical differences were found between HCC and cirrhotic patients. Nonetheless, its performance power demonstrated its discriminatory potential in detecting HCC with an AUROC of 0.84, 86% sensitivity, and 72% specificity [89]. To date, no prospective studies have assessed the predictive value of GDF15 in HCC detection or its role in non-viral hepatitis related HCC.

### 3.4. Vascular Endothelial Growth Factor (VEGF)

Besides its role as a potent angiogenic factor for vascular endothelial cells during HCC formation, as described above, VEGF has also been studied as a potential biomarker for HCC detection [90]. A retrospective Japanese study showed increased serum VEGF levels in 59 HCV-related HCCs compared to 28 cirrhotic and 37 non-cirrhotic HCV controls. The diagnostic performance of VEGF was better than other commonly used biomarkers, such as alpha-fetoprotein (AFP). This study showed an AUC for VEGF of 0.98 and 0.71 for AFP (sensitivity: 0.86 and 0.75 for VEGF and AFP, respectively) [91]. In contrast, a comparable study from Egypt on HCV-related HCC patients did not detect serum VEGF differences with the HCV control group [92]. These conflicting findings may be explained by ethnic background differences and HCV genotypes. However, both studies were relatively small and larger cohorts to further clarify these ambivalent results are needed. Interestingly, a more recent longitudinal study from our group identified serum VEGF as 1 out of 12 immune mediators to be increased in a group of 13 European chronic HCV patients who developed de novo HCC within 18 months of HCV therapy compared to matching controls. In our study, the performance has an AUROC value of 0.8 [93]. However, these findings were obtained in a small cohort, and in co-measurement with other immune analytes. 

## 4. Cytokines Related to Advanced HCC

The definition of advanced disease in HCC could be evaluated by a variety of factors. Of these, the BCLC staging is endorsed by the major liver disease societies and has been well validated [94,95].The BCLC staging system denotes stage C as advanced stage and stage D as terminal stage [96]. Multiple cytokines and stimulatory molecules are associated with the risk for advanced disease in patients with HCC. 

### 4.1. Interleukin-10 (IL-10)

IL-10 is a potent anti-inflammatory cytokine [97]. Produced by most activated immune cells, including monocytes and macrophages, IL-10 acts by reducing the production of inflammatory mediators, inhibiting antigen presentation, and suppressing numerous other immune parameters [98,99]. Its role in viral infections is well documented, but its role in HCC is less clearly understood. A recent meta-analysis showed that IL-10 levels in HCC patients are increased compared to cirrhotic patients and healthy controls, but not to patients with viral hepatitis, thereby adding complexity to the interpretation of IL-10 data for HCC [20]. One study of 67 individuals with resectable HCC provided evidence of worse post-operative outcomes in patients who had IL-10 level >12 pg/mL [100]. The role of IL-10 in unresectable HCC has also been researched. One retrospective study of 74 patients with unresectable HCC demonstrated that serum IL-10 levels acted as a negative prognostic factor with a significantly shorter median survival (3 months compared to 12 months; *p* < 0.02) [101]. In a larger series of 222 subjects with unresectable HCC (predominantly HBV related), the overall survival of patients with high serum IL-10 levels was significantly worse than that of the low IL-10 group (hazard ratio [HR] 2.2) [102]. Among those with advanced disease (BCLC stage C), individuals with high IL-10 levels had an overall survival of 3.5 months, much shorter than those with lower IL-10 levels at 10.2 months [102].

### 4.2. Interleukin-37b (IL-37b)

IL-37b is the largest of the five different isoforms of IL-37 (designated IL-37a-e) [103,104]. This cytokine is secreted by monocytes, macrophages and epithelial cells, and suppresses proinflammatory cytokine production and block EMT via downregulation of IL-6/STAT3 signaling [105,106]. Moreover, in vivo experiments with recombinant IL-37b in mice showed lower tumor volume than in untreated controls [107]. In a study conducted in HBV-related HCC patients, IL-37b serum levels had an inverse correlation to the prognosis of advanced HCC. Subjects in the high IL-37b group had better overall survival (38.3 vs. 28.9 months) and disease-free survival (33.5 vs. 23.6 months, DFS) [106]. Similarly, multivariate analysis showed high IL-37b expression in HCC tissues to be associated with greater overall survival and DFS in a largely HBV-HCC cohort [107]. These findings in HCC as well as the attenuated production and expression of IL-37b in metastatic cancers suggest an involvement for IL-37b in the signaling pathways that modulate metastasis, suggesting a potential role in histopathologic prognostication [108].

### 4.3. CC Chemokine Ligand 20 (CCL20)

CCL20 (also known macrophage inflammatory protein-3 alpha) interacts with CC chemokine receptor 6 (CCR6), resulting in chemoattraction of immune cells to inflammation sites. CCL20 has been shown to display a variety of roles in overall inflammation, rheumatoid arthritis and several cancers [109,110]. In vitro and in vivo assays have highlighted a role for the CCL20-CCR6 axis in inducing HCC proliferation, growth and invasion [111]. Moreover, in a study analyzing 33 specimens from 22 subjects, overexpression of CCL20 was found in tumors supporting a role in hepatocarcinogenesis [112]. Other studies have demonstrated a high-level expression of CCL20 and its receptor CCR6 in HCC and colorectal cancer liver metastasis, therefore indicating its involvement in tumor invasion, angiogenesis and progression of hepatic malignancies105. However, only one small study with 11 HCC patients reports a significant association between CCL20 expression and tumor grading (TNM stage 3 vs. 2) [113]. A study with 293 subjects with HCC, found that tumor-infiltrating regulatory T cells could be selectively recruited to the tumor through the CCR6-CCL20 axis. This study showed that the expression of CCL20 in the tumor was positively correlated with the number of tumor-infiltrating regulatory T cells. Importantly, the increased numbers of tumor-infiltrating regulatory T cells predicted poorer prognosis in HCC patients [114].

## 5. Cytokines Related to HCC Systemic Therapy Response

Most patients present at advanced HCC stages where treatment options are restricted to recently approved immune-checkpoint inhibitors or kinase inhibitors, such as sorafenib, regorafenib, and lenvatinib among others, all which block tumor growth and angiogenesis pathways [115]. Thus, evaluation of cytokines associated with these carcinogenic processes may help identify prognostic factors in response to therapy. In recent years, immune therapy has become a key player in the systematic treatment of HCC with several combinations approved for first- and second-line treatment. Moreover, the success of bevacizumab, a VEGF antibody, in combination with atezolizumab, a PD-L1 inhibitor, for the treatment of advanced HCC highlights the potential role of these immune players in the treatment of HCC [116]. Thus far, most studies addressing biomarkers for response to immune therapy have focused on immune checkpoint markers (i.e., PD-1, CTLA-4), mutational burden, and circulating DNA [117,118]. Due to the lack of studies involving cytokines in response to immune therapy we do not focus on that aspect in this review, but provide a general overview. Indeed, we mainly discuss cytokines with potential clinical utility under sorafenib as there is an extended body of work available, although new data are becoming rapidly available for other forms of systemic therapy (Table 1) [119,120].

### 5.1. Interleukin-6 (IL-6)

In the context of advanced HCC, a study on 128 sorafenib-treated HCC patients (93% Child–Pugh class A) divided over a discovery and validation cohort evaluated the prognosis value of pretreatment serum IL-6 levels. In both cohorts, a high pretreatment serum IL-6 level (cut-off: 4.28 pg/mL) was an independent predictor of poor overall survival [121]. However, there was no association with sorafenib effectiveness as progression-free survival and time to progression was similar irrespective of pretreatment IL-6 levels. Moreover, IL-6 pretreatment levels did not associate with macrovascular invasion or extrahepatic spread [121]. Although promising, further studies, which are currently being conducted, are needed to solidify the role of IL-6 in response to therapy in HCC. Interestingly, recent studies in cellular models have described decreased resistance to sorafenib by inhibiting IL-6-related pathways [126].

### 5.2. Angiopoietin-2 (ANG-2)

ANG-2 is almost exclusively produced by epithelial cells and acts as a key regulator in vessel maturation supporting the activities of other endothelial-acting cytokines [6,127,128,129]. In the SHARP study—the first randomized placebo-control trial to evaluate the role of sorafenib in advanced HCC, as well as the prognostic value of several cytokines—higher pretreatment ANG-2 levels were associated with lower overall survival, both in the overall cohort (*n* = 602) as well as in the sorafenib arm (*n* = 299). However, treatment interaction analysis found no correlation with sorafenib-associated survival. Nonetheless, patients who experienced an increase in plasma ANG-2 levels at week 12 were found to have shorter overall survival and time to progression compared to those patients with no increase in plasma levels [122]. One year later, the Okayama Liver Group (Japan) conducted a retrospective study followed by a longitudinal study on serum cytokines in two distinct sorafenib-treated advanced HCC cohorts (predominantly Child Pugh A) [123,125]. Similar to the SHARP study, increased pretreatment ANG-2 levels were associated with shorter overall survival [123,125]. Furthermore, patients with progressive disease showed increased ANG-2 levels at the start of therapy, compared to those with non-progressive disease, although the difference was not significant when the authors evaluated ANG-2 in a prospective cohort, possibly due to a reduced number of patients [123,125]. ANG-2 levels, however, only increased in patients with progressive disease during follow-up [125].

### 5.3. Hepatocyte Growth Factor (HGF)

In vitro studies and animal models have shown the HGF can have either promoting or a suppressive role in the development of HCC [130]. In the SHARP study, higher pretreatment plasma HGF levels were an independent prognostic factor for lower overall survival in the overall cohort and sorafenib arm [122]. Interestingly, lower HGF levels at the start of therapy tended to yield greater benefit from sorafenib in overall survival and time to progression. Furthermore, a decrease in median HGF plasma levels at 12 weeks, seen only in the sorafenib group, was associated with longer time to progression but not overall survival in the treatment arm [122]. Likewise, the Okayama Liver Group showed pretreatment levels of serum HGF to be a potential independent predictor of overall survival in prospective cohort albeit upon multivariate testing significance was lost [123]. Moreover, HGF pretreatment levels were increased in patients with progressive disease compared to non-progressive disease, albeit only significant in the retrospective cohort [123].

### 5.4. Vascular Endothelial Growth Factor (VEGF)

As a key cytokine driving angiogenesis, multityrosine kinase inhibitors such as Sorafenib target VEGF signaling. In addition to ANG-2 and HGF, the SHARP study also evaluated VEGF as a prognostic marker. Similar to the ANG-2, higher VEGF pretreatment levels were associated with lower survival. However, its prognostic value was not translated in the Sorafenib arm [122]. Interestingly, mean plasma VEGF were significantly increased in the Sorafenib vs. placebo group [122]. Moreover, a retrospective study conducted by the Okayama Liver group on HCC patients treated with Sorafenib found that VEGF levels were increased in patients who later experienced disease progression vs. non-disease progression [123]. In addition, and in concordance with data revealed by the SHARP study, elevated VEGF levels at baseline correlated with reduced overall survival and progression free-survival. However, multivariate analysis failed to identify VEGF as a prognostic factor for overall survival [123]. This observation was later confirmed by the same study group in a prospective cohort of Sorafenib-treated HCC patients [125]. Interestingly, Tsukiya et al. showed that a 5% decrease in plasma VEGF levels at 8 weeks from baseline was an independent prognostic factor associated with 1-year survival after Sorafenib treatment in a small cohort of HCC patients (*n* = 63) [131].

## 6. Cytokines Associated with Response to Immune Checkpoint Inhibitor Therapy

In recent years, immune checkpoint inhibitors (ICI) have expanded the treatment options for HCC. These agents target the co-inhibitory cell signals via the programmed death ligand/receptor (PD-L1/PD-1) and/or cytotoxic T-lymphocyte associated antigen-4 (CTLA-4) [132]. Despite the promise shown by these agents in clinical trials, the response rates in clinical practice may be less than 40%, hence the need for predictors of response to ICI treatment [133]. Most of the studies and data regarding biomarkers for ICI response are very limited and recent. We therefore highlight below some of the studies in the field. Nonetheless, further research and confirmation is needed for those markers to be considered in clinical practice. Pretreatment levels of PD-1/PD-L1 are well observed to predict response to ICI therapy, as well as the risk of acute cellular rejection when used in liver transplant recipients [134,135]. Beyond PD-1/PD-L1, the use of other peripheral biomarkers in the prediction of response to ICI is somewhat limited, but there have been a few biomarkers of interest with early assessment, including OPN, T-cell immunoglobulin and mucin domain-containing-3 (TIM-3), V-domain immunoglobulin suppressor of T-cell activation (VISTA), and C-C motif chemokine 5 (CCL5/RANTES) [136,137,138]. In a study on the effect of OPN and the colony-stimulating factor-1/receptor (CSF1/CSF1R) pathway in HCC-bearing mice, Zhu et al. noted that anti-PD-L1 and CSF1R inhibition in mice with high OPN elicited potent anti-tumor activity and prolonged survival [136]. Furthermore, in a trial using a discovery cohort of 21 patients and a validation cohort of 61 patients with multiple cancer types (31% HCC), the combined expression of soluble PD-L1 as well as CCL5/RANTES was helpful in predicting improved disease control (AUC 0.722, p 0.003) [138]. Finally, smaller studies of patients with HCC on ICI therapy have suggested a potentially predictive role of baseline levels of inflammatory cytokines, such as transforming growth factor-beta (TGF-β) [139]. The above-mentioned studies are either in animal models, in very small cohorts, or retrospective assessment of public databases, and larger studies should be performed to better understand the roles of these markers in ICI for HCC.

## 7. Conclusions

Cytokines are complex immune molecules active in a variety of diseases, including cancer. In HCC, cytokines have been found to have a role in different aspects of tumor formation and detection. This review intended to present cytokines of clinical relevance and their interconnection with different aspects of HCC, highlight their contribution in tumor promotion as well as in detection and response to therapy. As the need for soluble HCC biomarkers that are simple to measure continues, cytokines represent an attractive solution since their measurement only requires basic laboratory equipment. However, the immune dysregulation underlying the different liver diseases that give rise to HCC (i.e., chronic viral infections, NAFLD) challenges the implementation of these cytokines as reliable biomarkers. Recent studies have aimed to evaluate a combination of different cytokines in a signature fashion in HCC of specific underling etiologies, improving their potential as important players in HCC surveillance. Advances in measurement techniques, stratification of cohorts, understanding of specific roles by cytokines in HCC, and possibly biomarker combination/s with tumor specific markers will further the path to their potential use in clinical practice.

## Figures and Tables

**Table 1:** Evaluation of serum biomarkers with potential prognostic value in systemic HCC therapy.

THERAPY	N PATIENTS	TYPE OF STUDY	EVALUATED CYTOKINE/S—BIOMARKERS	OUTCOMES	REFERENCES
SORAFENIB	128	Retrospective	IL-6	OS, PFS and TTP	Shao et al., 2017 [121]
SORAFENIB	299	Randomized–controlled	ANG-2, EGF, bFGF, VEGF, sVEGFR-2, sVEGFR-3, HGF, and s-c-KIT, IGF-2	OS, TTP	Llovet et al., 2012 [122]
SORAFENIB	120	Retrospective	ANG-2, FST, G-CSF, HGF, Leptin, PDGF-BB, PECAM-1, and VEGF	OS, PFS	Miyahara et al., 2013 [123]
SORAFENIB	91	Retrospective	TGF-β	OS, PFS	Lin et al., 2015 [124]
SORAFENIB	80	Prospective	FST, G-CSF, HGF, Leptin, PDGF-BB, PECAM-1, ANG-2, VEGF	OS, PFS	T. Adachi et al., 2019 [125]
LENVATINIB	41	Retrospective	aFGF, bFGF, FGF-23, VEGF-R3, VEGF-C, VEGF-D, EGF, Fas, FasL, IL-1R2, PDGF-BB, TSP-2, Ang-1, ANG-2, Tie-2, CXCL8, HGF, Neuropilin-1, c-MET, HGF, IFN-β	OS, PFS, PD	Ono et al., 2020 [120]
REGORAFENIB	332	Randomized–controlled	294 biomarkers (DiscoveryMAP)	OS, TTP	Teufel et al., 2019 [119]

Abbreviations: IL-6, interleukin-6; ANG-1/2, angiopoietin-1/2; EGF, epidermal growth factor; VEGF, vascular endothelial growth factor; sVEGFR, soluble VEGF receptor; IFN-β, interferon beta; HGF, hepatocyte growth factor; s-c-KIT, soluble c-KIT, IGF-2, insulin-like growth factor -2; FST, follistatin; G-CSF, granulocyte colony stimulating factor; PDGF-BB, platelet-derived growth factor BB; PECAM-1, platelet endothelial cell adhesion molecule; aFGF, acidic fibroblast growth factor; bFGF, basic fibroblast growth factor; FGF, fibroblast growth factor; IL-1R2, interleukin-1 receptor 2; TSP-2, thrombospondin-2; Tie-2, tyrosine-protein kinase receptor Tie-2; CXCL8, chemokine (C-X-C motif) ligand 8. OS, overall survival; PFS, progression-free survival; TTP, time to progression; PD, (early) progression disease.
